# Building Markov state models with solvent dynamics

**DOI:** 10.1186/1471-2105-14-S2-S8

**Published:** 2013-01-21

**Authors:** Chen Gu, Huang-Wei Chang, Lutz Maibaum, Vijay S Pande, Gunnar E Carlsson, Leonidas J Guibas

**Affiliations:** 1 Institute for Computational and Mathematical Engineering, Stanford University, Stanford, CA 94305, USA; 2 Department of Chemistry, University of Washington, Seattle, WA 98195, USA; 3 Department of Chemistry, Stanford University, Stanford, CA 94305, USA; 4 Department of Mathematics, Stanford University, Stanford, CA 94305, USA; 5 Department of Computer Science, Stanford University, Stanford, CA 94305, USA

## Abstract

**Background:**

Markov state models have been widely used to study conformational changes of biological macromolecules. These models are built from short timescale simulations and then propagated to extract long timescale dynamics. However, the solvent information in molecular simulations are often ignored in current methods, because of the large number of solvent molecules in a system and the indistinguishability of solvent molecules upon their exchange.

**Methods:**

We present a solvent signature that compactly summarizes the solvent distribution in the high-dimensional data, and then define a distance metric between different configurations using this signature. We next incorporate the solvent information into the construction of Markov state models and present a fast geometric clustering algorithm which combines both the solute-based and solvent-based distances.

**Results:**

We have tested our method on several different molecular dynamical systems, including alanine dipeptide, carbon nanotube, and benzene rings. With the new solvent-based signatures, we are able to identify different solvent distributions near the solute. Furthermore, when the solute has a concave shape, we can also capture the water number inside the solute structure. Finally we have compared the performances of different Markov state models. The experiment results show that our approach improves the existing methods both in the computational running time and the metastability.

**Conclusions:**

In this paper we have initiated an study to build Markov state models for molecular dynamical systems with solvent degrees of freedom. The methods we described should also be broadly applicable to a wide range of biomolecular simulation analyses.

## Background

The simulation of biological processes at the molecular scale has the potential to give insight into a wide range of properties and phenomena that are important to science, engineering, and medicine -- with protein folding, or mis-folding, being perhaps the most famous example [1,2]. Indeed, simulations can give, in principle, atomic-level detail with great temporal precision over a wide range of application areas, thus greatly complementing and expanding on what one can currently do experimentally. Today, with powerful individual processors, large computer clusters, as well as with very large distributed clusters of processors, one can routinely generate massive quantities of simulation data for a given phenomenon of interest, often in full-atomic detail along many trajectories.

There is an increasing need to mine such massive data sets in order to gain insight into the fundamental phenomena under study. From these data sets, the goal is to understand at some more macroscopic level the structure of the paths taken during the simulation. The key challenge facing dynamical simulation on the molecular scale is to overcome the gap between the timescales where interesting biologically relevant conformational changes occur (typically microseconds or even longer) and those we can simulate at atomic resolution (typically nanoseconds). The length of atomic simulations is limited by the need to take small time steps, which is determined by the high frequency motions.

### Markov state models

To meet such a challenge, a lot of recent effort has been devoted to constructing stochastic kinetic models, often in the form of *discrete-time Markov state models (MSMs)*, from relatively short molecular dynamics simulations [3-11]. These models are built from short timescale simulations and then propagated to extract long timescale dynamics. The MSMs partition configuration space into a number of distinct states, called *metastable states*, such that the intra-state transitions are fast but the inter-state transitions are slow. Such separation of timescales ensures that the model is Markovian, in that the probability of being in a given state at time *t *+ Δ*t *depends only on the state at time *t*.

In a MSM, the time evolution of a vector representing the population of each state can be calculated as *P*(*nτ *) = [*T*(*τ*)]*^n^P*(0), where *P*(*nτ*) is a vector of state populations after *n *time steps and *T*(*τ*) is the column-stochastic transition probability matrix with lag time *τ *(simulation time step). Note that any model is Markovian for a sufficiently long lag time *τ*, because the system is able to converge to an equilibrium distribution from any arbitrary initial distribution after one lag time. The key point is to build a model with a lag time that is shorter than the timescale of the process of interest with a reasonable number of states.

To build such dynamical models, it is necessary to map out the dominant long lived, kinetically metastable states and then determine the rates for transitioning between these states. A few different approaches have been developed to generate good state decompositions. If the low-dimensional manifold containing all the slow degrees of freedom is known a priori, then the configuration space can be partitioned into free energy basins to define these metastable states, such as by examination of the potential of mean force [10-14]. Without this prior knowledge, some attempts have turned to conformational clustering techniques which assume that geo-metrically distinct clusters may also be kinetically distinct [15-18].

In [4], Chodera et al. proposed a first algorithm that can automatically discover kinetically metastable states for the construction of MSMs. They use a geometric clustering algorithm to split the configuration space into a large number of small microstates, and then lump them into kinetically distinct macrostates. Later, Bowman et al. developed an open source software package MSMBuilder based on this framework [6]. The software provides tools for clustering data based on geometric relationships and for constructing and manipulating MSMs based on this initial clustering. It also includes tools for verifying that the resulting model is Markovian as well as analyzing and visualizing the model. There are also several recent works developed related to these methods [7-9].

### Solvent degrees of freedom

Since the dynamics of biological macromolecules are usually coupled with the surrounding solvent, many molecular simulations involve both a solute and a solvent (typically water). Some previous works have shown the necessary of accounting for the solvent structure to accurately characterize the dynamics and free energy landscape of the biological macromolecule systems, such as the RNA hairpin-loop motif [19], alanine dipeptide [20] and the BphC enzyme [21]. In this setting, both solute and solvent atoms are placed in a box and then move following some predefined force field, yielding a sequence of snapshots of the atom positions. The number of atoms is kept constant in this process.

Although people have recognized that solvent coordinates may be critical in some phenomena [19-25], in the step of data analysis people often assume configurations lie exclusively in the configuration space of the macromolecule, and simply ignore the solvent information. For example, in [4], it presume that de-correlation of momenta and reorganization of the solvent is faster than the process of interest. One difficulty in dealing with solvent degrees of freedom is the large number of solvent molecules in a system (typically thousands). Besides, it also requires to account for the indistinguishability of solvent molecules upon their exchange. One impressive work in this direction is [22], which used a generic neural network model to identify reaction coordinates from a database of candidate variables including water related ones. However, to use this approach researchers have to define the candidate variables. Furthermore, the result from the neural network model may not be easy to interpret, which is a drawback as a data exploration tool.

In this paper, we propose to generalize the current methods to include the solvent degrees of freedom. We first present a new distance metric which encodes the solvent information in molecular configurations, and then incorporate it into the construction of MSMs. Finally we apply our method to several biological model systems and assess its performance.

## Methods

Many of the dynamical systems which occur in biochemistry take place in very high dimensional spaces. Our main goal is to develop techniques to obtain the simplest kind of qualitative information about high-dimensional molecular dynamical systems. Perhaps the most significant piece of information one has about the data set is the distance metric which specifies the distances between pairs of points (molecular configurations). For macromolecules, a commonly used metric for estimating the distance between two molecules is the *RMSD distance*, defined as the root mean squared deviation of the Cartesian coordinates of heavy atoms in the molecules after a minimizing rigid body translation and rotation alignment [26,27]. In this section, we design a new distance function for comparing the solvent profiles, and then use it to construct MSMs with solvent degrees of freedom.

### Distance functions

In molecular simulations, a system consists of both a solute (macromolecule) and a solvent (water). Suppose the solute structure contains *m *atoms, and the solvent involves *n *water molecules. We denote *X *= {*x*_1_, *x*_2_, ..., *x_m_*} as the set of solute atoms, and *Y *= {*y*_1_, *y*_2_, ..., *y_n_*} representing the set of solvent atoms. (For water molecules, we only record the oxygen atom at the vertex and ignore two hydrogen atoms at the tips, so each *y_i _*corresponds to the oxygen atom of a water molecule). Then, the results of the simulations become sequences of point sets {*X, Y*}, which are obtained by sampling at random from the configuration space and then following the trajectory for a certain time interval.

We first point out two properties when comparing different configurations {*X, Y*}:

• *m *≪ *n *-- typically the number of solute atoms is less than 100, while there can be thousands of solvent molecules in a system.

• {*y_1_, y_2_*, ..., *y_n_*} are indistinguishable upon their exchange -- when considering the interaction between the solute and the solvent, we do not care about the identities of *Y*. In other words, two configurations are considered as the same if they only differ by a permutation of solvent molecules.

To address the indistinguishability of solvent molecules upon their exchange, one may consider methods that compute the optimal matching between the solvent molecules, such as minimum cost flow [28], or the Hungarian algorithm [29]. However, these matching based algorithms would require *O*(*n*^3^) time, which is slow for systems with thousands of solvent molecules. The computational cost can be reduced if we only focus on solvent molecules around the solute, such as its *k*-nearest neighbors. However, this solution is not stable because a small perturbation in the configuration may cause the set of *k*-nearest neighbors to vary a lot.

We present a new distance function that measures the geometric similarity between different configuration. The idea is we compute some signatures/descriptors *f*(*X, Y*) that compactly summarize the high-dimensional data sets {*X, Y*}, and then define the distances using these signatures. As mentioned above, we would like the signature *f*(*X, Y*) to satisfy the following properties:

1. *f*(*X, Y*) is continuous in the input space *X *and *Y*, so a small perturbation of the system does not change the signatures too much.

2. *f*(*X, Y*) is symmetric in *Y *= {*y*_1_, *y*_2_, ..., *y_n_*}, so the solvent molecules are indistinguishable upon their exchange.

3. *y_i_*'s far from *X *have less weights in *f*(*X, Y*), because these solvent molecules have little impact to the solute.

To meet these properties, we define the signature *f*(*X, Y*) as follows. Given a point *x *∈ *X*, we transform the space using a Gaussian kernel $K\left(x,y\right)=\text{exp}\left(-\frac{||x-y|{|}^{2}}{2\sigma^{2}}\right)$, where ||*x *- *y*|| is the Euclidean distance between points *x *and *y*, so that *y_i_*'s far from *X *become less important. We then define the signature of a single point *x *relative to space *Y *as $f\left(x,Y\right)=\overset{n}{\underset{i=1}{\sum }}K\left(x,y_{i}\right)$. By summing up all kernels *K *(*x, y_i_*), the result is invariant under permutations of solvent molecules. Finally, we define *f*(*X, Y*) as a signature vector {*f*(*x*_1_, *Y*), *f*(*x*_2_, *Y*), ..., *f*(*x_m_, Y*)}, which takes *O*(*mn*) computation time.

Intuitively, the signature vector *f*(*X, Y*) summarizes the solvent distribution around each solute atom. We then define the distance between two configurations simply as the Euclidean distance between their signature vectors. In fact, there are various choices of functions that can satisfy these properties (1-3), while the one we proposed here is simple and fast to compute.

### Constructing Markov state models

In this section, we integrate the solvent information into the construction of MSMs. We will follow and extend the methods described in [4,6]. Basically, these approaches has two steps -- a *split *step to reduce the size of the data set based on geometric shapes, and then a *lump *step to incorporate kinetic information from trajectories.

#### Splitting

Modern computer simulations can easily generate data sets with millions of configurations, making analysis of these massive data sets computationally challenging. An important method for shrinking the data sets is to apply a clustering algorithm to obtain a family of clusters (microstates) of much smaller size than the original data set. Here each cluster should be small enough to ensure that the intra-state transitions between configurations in the same cluster are fast.

In the split step, all *N *configurations (10^4 ^- 10^7^) are grouped into *K *microstates (10^2 ^- 10^4^) based on their structural similarity. Due to the large size of the data set, it is more practical to apply a fast geometric clustering algorithm, such as the *k*-center or *k*-medoid algorithm with *O*(*KN*) time complexity [30,31]. Another important factor is the choice of distance functions in these clustering algorithms. In the traditional solute-based models, the RMSD distance is often used as a standard metric to measure the structural similarity. With the distance function we defined between solvent configurations, we are able to identify solvent-based metastable clusters. Furthermore, we may combine these two distance functions together to build a model with both solute and solvent information.

Suppose we want to build a model with *K *microstates, we first group all *N *configurations into $\left\lceil \sqrt{K\;}\right\rceil$ solute clusters using the RMSD distance, and then independently group all configurations into $\left\lceil \sqrt{K\;}\right\rceil$ solvent clusters using the distance based on solvent signatures. In the next step, we consider two configurations to be in the same microstate if and only if they are assigned to both the same solute cluster and the same solvent cluster, and thus there are totally ${\left\lceil \sqrt{K\;}\right\rceil }^{2}$ states at the end. Note that some states might be empty if there is no configuration assigned to their corresponding {solute, solvent} cluster pairs. In this case, we may increase the number of solute/solvent clusters a little bit larger to make sure that we have at least *K *non-empty states. (An alternate solution is to group all configurations into $\left\lceil \sqrt{K\;}\right\rceil$ solute clusters first, and then generate $\left\lceil \sqrt{K\;}\right\rceil$ solvent clusters for configurations within each solute cluster independently, instead of generating $\left\lceil \sqrt{K\;}\right\rceil$ global solvent clusters.) Finally, we form the *K *microstates by simply merging the smallest states (this step can be skipped if we do not need to form exactly *K *microstates).

More generally, we can generate *K*_1 _solute clusters and *K*_2 _solvent clusters (with *K*_1_*K*_2 _≥ *K*), and then combine them into *K *microstates. In fact, the traditional solute-based model can be seen as a special case where *K*_2 _= 1, and the solvent-based model is a special case where *K*_1 _= 1. Note that in this case, the running time for geometric clustering becomes *O*((*K*_1 _+ *K*_2_)*N*). By setting $K_{\text{1}}=K_{\text{2}}=\sqrt{K\;}$, we achieve the optimal running time $O\left(\sqrt{K}N\right)$ -- which is much faster than *O*(*KN*) time for large *K *(because we are generating hundreds/thousands of microstates).

#### Lumping

Because the clustering algorithms do not produce clusters of any particular uniform shape or size, we have lost the original metric information after the split step. What one retains, however, is the computation of probabilities for transitioning from one microstate to another. This means that we retain a coarse version of the dynamics. In the next step, these microstates are lumped into macrostates based on their kinetic transitions in the trajectories. Since this step does not consider solute/solvent information about configurations, we simply follow the same approach described in [4].

In the lump step, the *K *microstates are grouped into *L *macrostates (< 10^2^) so as to maximize the *metastability*. The metastability *Q *of a decomposition into *L *macrostates is defied as the trace of its transition probability matrix $Q=\overset{L}{\underset{i=1}{\sum }}T_{ii}\left(\tau \right)$. Intuitively, a poor decomposition would result in a small *Q*, as trajectories started in some states exit rapidly; conversely, a good decomposition with strongly metastable states would result in a large *Q*, as trajectories remain in each state for long times.

In the original approach, a simulated annealing algorithm [32] is used to optimize the metastability in lumping. The algorithm starts with an arbitrary initial solution that assigns *K *microstates into *L *macrostates. In each step, a microstate is selected uniformly at random, and reassigned to a new random macrostate (the new solution is rejected if a macrostate becomes empty after this change to ensure that there are *L *macrostates). If the new solution has a larger metastability *Q*' than the old solution *Q*, the new solution is accepted; otherwise it is accepted by a probability of $\text{exp}\left(\frac{Q^{\prime }-Q}{T}\right)$, where *T *is a temperature parameter which is set to be the inverse of the step number. The allowance for these "downhill" moves can potentially save the method from becoming stuck at local optima.

## Results and discussion

The method we described here would be generally applicable to a wide range of biomolecular simulation analyses. In this section, we pick several examples and test the performance of our method in these different models.

### Solvent-based clusters

We first apply our method to a small alanine dipeptide system, which has been used as an example in the MSMBuilder [4,6]. We pick a 5 nanoseconds trajectory of alanine dipeptide in explicit water, with a frame rate of 1 picosecond.

**Figure 1:**
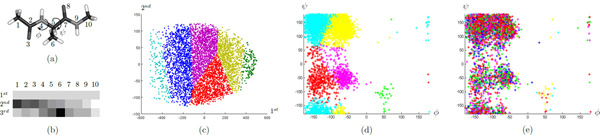
**Alanine dipeptide**. (a) Solute structure. (b) Top 3 PCA directions for signature *f*(*X, Y*) (*σ *= 1). (c) A solvent-based state decomposition mapped to the PCA space. (d) A solute-based state decomposition on the torsion angles map. (e) Projection of solvent-based clusters onto the torsion angles map.

In this model, the solute structure Ace-Ala-Nme consists of 22 atoms and the solvent contains 885 H_2_O. For each configuration, we extract 10 solute atoms *X *= {*x*_1_, *x*_2_, ..., *x*_10_} consisting of all heavy atoms on the backbone chain (see Figure 1(a)), and also *Y *= {*y*_1_, *y*_2_, ..., *y*_885_} representing the water molecules. We next reduce the dimensionality of this point set {*X, Y*} by computing its signature *f*(*X, Y*).

Intuitively, the signature vector *f*(*X, Y*) summarizes the solvent distribution around the solute. To see this, we map the signatures of all configurations onto a lower dimensional space using the principle component analysis (PCA) [33]. Figure 1(b) shows the top three PCA directions for *f*(*X, Y*), where the colors represent weights for each dimension. The first principle component is basically the average of *f*(*x_i_, Y*) at all solute atoms, which represents the amount of water around the whole solute structure. The second principle component distinguishes the two ends of the backbone chain, which tells us whether the water molecules are gathered on the left side or the right side. Furthermore, the third principle component distinguishes the two ends and the middle part, for example in the case when the two ends are folded close to each other. A six-states decomposition for all solvent signatures using the *k*-center clustering is shown in Figure 1(c), where the space is partitioned based on these PCA directions.

In protein backbone geometry, it is known that the torsion angles *ϕ *and *ψ *are the primary degrees of freedom of the solute structure. (The solvent coordinates have been shown to be the next most important degrees of freedom in this dynamical system [20,22].) For example, Figure 1(d) shows a five-states decomposition using the *k*-center clustering with RMSD distances, projected onto the (*ϕ, ψ*) torsion angles map (similar to the manual state decomposition described in [14]). However, these solute-based clusters are very different from those solvent-based clusters -- if we project the solvent clusters onto the torsion angles map, they no longer show a clustering behavior (see Figure 1(e)). This also motivated us the construction of the combination model which integrates both solute and solvent information, as described in the splitting section.

In the above alanine dipeptide example, the solute structure is small and may in some sense be considered as a convex object, because the water molecules rarely enter the region inside the solute structure. We next turn to another example of carbon nanotube in water, whose solute atoms form a very concave structure. Because this model simulates water molecules going in and out of a carbon nanotube, it is a good test of whether the solvent distribution inside the solute structure can be captured by our method.

**Figure 2:**
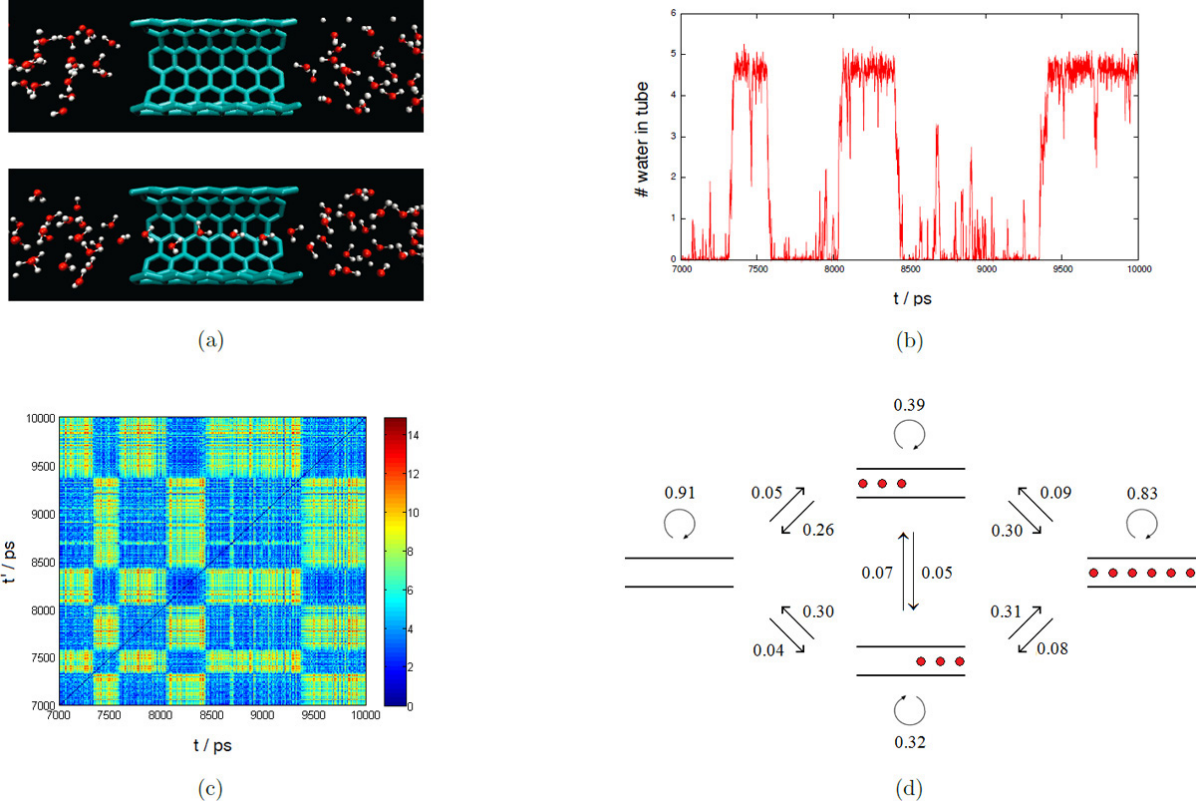
**Carbon nanotube**. (a) Empty and full metastable states. (b) Water number inside the nanotube. (c) Pairwise distance matrix between solvent signatures (*σ *= 1/3). (d) MSM with 4 states.

We have a 10 nanoseconds trajectory of carbon nanotube in water, with a frame rate of 1 picosecond. The solute *X *consists of 144 fixed carbon atoms with a cylindrical nanostructure, and the solvent *Y *contains 951 H_2_O. In [23], it has been observed the spontaneous and continuous filling of a nonpolar carbon nanotube with a one-dimensionally ordered chain of water molecules, and a minute reduction in the attraction between the tube wall and water can dramatically affect pore hydration, leading to sharp transitions between empty and full states on a nanosecond timescale (see Figure 2(a)). This can also be verified using our method by computing the *water number *inside the nanotube, which we define as the integral of point signature *f*(*x, Y*) over the cylindrical region *V *inside the nanotube. Here we use a normalized Gaussian kernel $K\left(x,y\right)=\frac{1}{{\left(\sqrt{2\pi }\sigma \right)}^{3}}\text{exp}\left(-\frac{{∥x-y∥}^{2}}{2\sigma^{2}}\right)$. Note that the water number $\int_{V}f\left(x,Y\right)\text{dx}=\int_{V}\overset{n}{\underset{i=1}{\sum }}K\left(x,y_{i}\right)dx=\overset{n}{\underset{i=1}{\sum }}\int_{V}K\left(x,y_{i}\right)dx$. As *σ *→ 0, *K*(*x, y_i_*) converges to the Dirac delta function centered at *y_i_*, and thus ∫*_V_K*(*x, y_i_*)d*x *can be seen as an indicator function *I*(*y_i _*∈ *V*). So, the water number roughly counts the number of water molecules inside the nanotube, except that it is a continuous function. Figure 2(b) plots the water number inside the nanotube over a period of 3000 frames. In this figure, we can clearly see that the system transits between empty and full states, with fast intra-state transitions and slow inter-state transitions. (In a full state, there can be at most six water molecules inside the nanotube. Note that the leftmost and rightmost ones appear near the boundary of region *V*, so each of them contributes about 1/2 to the water number.)

However, the above computation of water number relies on the fact that the system dynamics depends on the distribution of water molecules inside the nanotube. In general, we have no prior knowledge about how to choose a proper region *V *of interest, but we can use the solvent signature *f*(*X, Y*) = {*f*(*x*_1_, *Y*), *f*(*x*_2_, *Y*), ..., *f*(*x_m_, Y*)} as a compact representation of the solvent distribution around the carbon nanotube. Figure 2(c) plots the pairwise distances between these solvent signatures, and we can see a notable block structure in this matrix -- it is easy to distinguish empty and full states, because the distances between empty/empty, or full/full states are small, while the distances between empty/full states are large. For example, if we apply the *k*-center clustering with *K *= 2, it returns two clusters correspond to these two main metastable states, with intra-state transition probabilities 0.96 (empty) and 0.94 (full) respectively.

Figure 2(d) shows a more refined model with *K *= 4. In addition to the empty and full stable states, it includes two transition states with much smaller intra-state transition probabilities. The centers of these two new clusters correspond to configurations in which the nanotube is left/right half-full. This implies that for a transition between the empty state and the full state, all water molecules inside the nanotube enter (leave) from either the left side, or the right side, but not simultaneously from both directions. (In Figure 2(a), we can see that the dipole moments of all water molecules inside the nanotube point to the left direction.) Furthermore, the inter-state transition probabilities between these two transition states are very small, which means it is unlikely that water molecules inside the nanotube in a left half-full state can shift into a right half-full state, and vice versa. Thus, the model we derived here coincides with the observations in [23].

### Comparing different models

We have defined three types of models in the construction of MSMs: (1) a solute-based model using RMSD distances, (2) a solvent-based model using solvent signatures, and (3) a combination model integrating both the them. In this section, we compare the performances of these different models. In particular, we use the metastability as a measure, which is also the objective function that we optimized in building MSMs.

**Figure 3:**
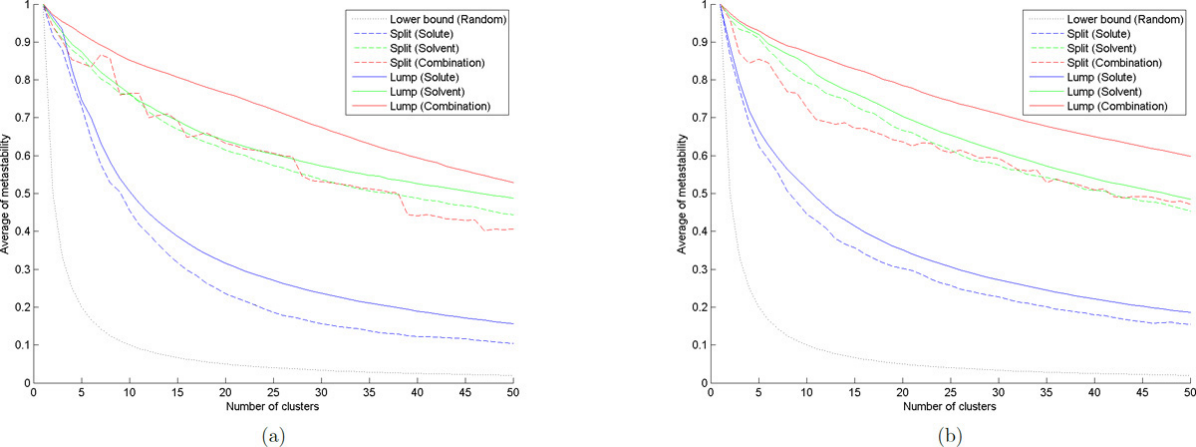
**Metastability of MSMs**. (a) Alanine dipeptide. (b) Benzene rings.

Figure 3(a) shows the experiment results for the alanine dipeptide model. To compare results with different number of states, we have normalized the metastability *Q *by the number of clusters (microstates/macrostates) as the vertical plot. The dotted line at the bottom shows a naive lower bound for any clustering algorithm -- if we randomly assign each configuration to one of the *K *clusters, then the expected average of metastability is 1/*K*.

For splitting, the *k*-center algorithm is used as the fast geometric clustering algorithm since it returns clusters with approximately equal radii [8]. The dashed lines show the results after the split step. In the solute-based model, we see that the average of metastability decreases very quickly as we increase the number of microstates, while the solvent-based model seems to be much more stable (this also implies the solvent changes slower than the solute in the alanine dipeptide system). The combination model performs close to the solvent-based model, however, the advantage is that it takes only $O\left(\sqrt{K}N\right)$ time, instead of *O*(*KN*) time.

For lumping, we first split all configurations into *K *= 100 microstates, and then lump them into *L *macrostates for each 1 ≤ *L *≤ 50. For each test case, we run the simulated annealing algorithm 100 times independently, and each run simulates for 10000 steps. The solution with the highest metastability sampled in any run is selected to define the lumping into macrostates. The solid lines show the results after the lump step, and the gap between solid and dashed lines corresponds to the improvement by simulated annealing. After incorporating the kinetic information, we see that the metastabilities for the solute-based model and the combination model are significantly improved, and thus the combination model gives the best result. The reason is that for solute configurations, there may exist structures which are geometrically close but are kinetically very different, because the deformation from one to another may needs to follow a long trajectory to avoid collisions between backbone links [9]. However, for solvent configurations, there are no such links between different water molecules (only H-O links within each water molecule), so solvent configurations that are geometrically close should also be kinetically close. Therefore, the gap in the solvent-based model is much smaller than those in the other two models.

**Figure 4:**
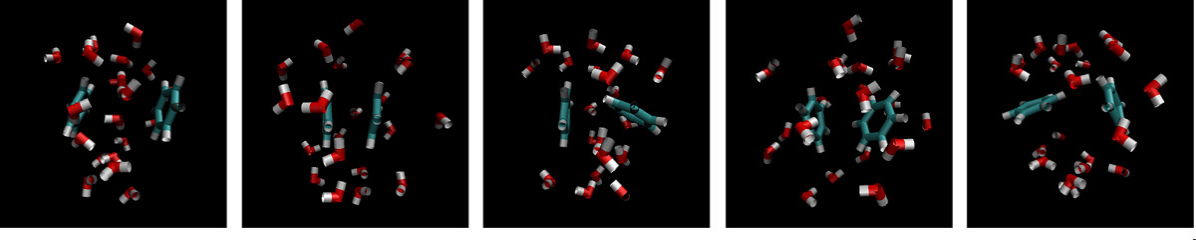
**Snapshots of different configurations in the benzene rings system**.

We have also verified this result on another data set for the collapse of benzene rings (see Figure 4), which simulates the dewetting and hydrophobic interaction in a biological system [24,25]. In this model, the solute consists of two separate hexagonal rings, each having 6 carbon atoms with 6 attached hydrogen atoms, and the solvent contains 2470 H_2_O. The system is simulated for 100 nanoseconds, with a frame rate of 2 picoseconds. The experiment results for this benzene rings model are shown in Figure 3(b), in which the performance is close to the previous alanine dipeptide model.

## Conclusions

In this paper we have initiated an study to build Markov state models for molecular dynamical systems with solvent degrees of freedom. We have introduced a Gaussian-based signature to compactly represent the solvent distribution in the configuration space, and incorporated this information into the construction of MSMs to identify metastable solvent clusters. We have also tested our method on several different biological data sets and find that our approach improves the existing methods both in the computational running time and the metastability. We believe that the methods we described would be more generally applicable to a wide range of biomolecular simulations.

## Competing interests

The authors declare that they have no competing interests.

## Authors' contributions

CG, HWC and LM executed this study and wrote the draft of this manuscript. VSP, GEC and LJG supervised this project.

## Declarations

The publication costs for this article were funded by NSF grant DMS 0900700.

This article has been published as part of *BMC Bioinformatics *Volume 14 Supplement 2, 2013: Selected articles from the Eleventh Asia Pacific Bioinformatics Conference (APBC 2013): Bioinformatics. The full contents of the supplement are available online at http://www.biomedcentral.com/bmcbioinformatics/supplements/14/S2.
